# Operator wellbeing as a translational endpoint: a dimension missing from the AI implementation framework in anesthesiology

**DOI:** 10.1007/s10877-026-01444-w

**Published:** 2026-06-06

**Authors:** Valentina Bellini, Matteo Panizzi, Elena Bignami

**Affiliations:** Anesthesiology, Critical Care and Pain Medicine Division, Department of Medicine and Surgery, University of Parma, Viale Gramsci 14, Parma, 43126 Italy

**Keywords:** Artificial intelligence, Anesthesiology, Wellbeing, Quality of work, VUCA, BANI

The narrative review by Baliga and Seshadri [1] offers a thorough account of the barriers separating AI research from clinical practice in anesthesiology. The authors map the translational gap across data quality, regulatory complexity, EHR interoperability, and alarm fatigue creating a genuinely useful taxonomy. One dimension, however, receives little attention in their framework: the impact of AI-assisted workflows on the wellbeing of anesthesia providers. We believe this warrants explicit consideration, and that it opens a pragmatic secondary implementation pathway that does not depend on the completion of multicentric prospective trials and hard regulatory constrains.

Anesthesiology is under considerable occupational strain. Burnout among anesthesiologists carries an estimated annual cost exceeding $4.6 billion in the United States alone, and workforce shortages are projected to worsen substantially over the coming decade [2]. Against this backdrop, the Anesthesiology 5.0 framework proposed by Loepke positions human-centricity not as a supplemental wellbeing initiative, but as a foundational design requirement for any sustainable integration of technology into clinical practice [2]. The relevance of this perspective to the translational challenge described by Baliga and Seshadri should not be underestimated: provider attrition and cognitive overload undermine the very institutional capacity required to implement and sustain AI tools.

A concrete illustration of what a lower-barrier implementation pathway might look like is provided by the experience of Hurley et al. [3], who deployed a ChatGPT-assisted scheduling tool to design six-month rotas for 27 anesthesia residents. The results were substantively favorable: 100% of first-preference annual leave requests were approved, on-call commitments were distributed equitably across all three clinical tiers (theatres, obstetrics, and critical care), and final leave decisions were returned within a single working day. No randomized trial was required. No regulatory clearance was sought. No EHR integration was necessary. The intervention targeted a specific, well-defined source of occupational friction — opaque and inequitable scheduling — that has direct links to resident burnout and morale [3]. This is precisely the category of problem that Loepke identifies as a “recurring microfriction” eroding daily professional life [2].

The cognitive dimension of workflow AI connects to patient safety as well as provider wellbeing. Weller et al. [4] have shown that communication failures contribute to 60–70% of serious clinical incidents, and that shared situational awareness within the anesthesia team is a critical determinant of both safety outcomes and staff experience. AI systems that process information continuously, without fatigue, are well positioned to support team situational awareness — provided they are designed to reduce cognitive demands on clinicians rather than generate additional alert burden [4].

The eleven-step implementation checklist proposed by Baliga and Seshadri [1] is a valuable contribution, but it is framed around bedside clinical AI tools and the evidentiary standards appropriate to them. We suggest that the framework would be more complete if it explicitly distinguished between two parallel implementation tracks: a high-barrier track for AI-based clinical decision-support tools requiring prospective multicentric outcome data, and a lower-barrier AI operational track targeting scheduling, administrative load, and cognitive ergonomics, where the primary endpoint is measurable provider wellbeing and workforce sustainability. The two tracks are not in competition — they share infrastructure, build institutional AI literacy, and can be pursued concurrently.

Recognizing provider wellbeing as a legitimate and measurable endpoint for AI implementation may accelerate adoption along pathways that purely clinical or economic arguments have thus far failed to achieve. An AI tool that reduces the workload or improves the quality of the work is a tool that it’s worth invest on as much as a clinical decision system. The translational gap will narrow faster if the human capacity to implement, sustain, and trust AI tools is treated as a prerequisite — not an afterthought.

## Data Availability

No datasets were generated or analysed during the current study.
